# Inhibiting Cytoprotective Autophagy in Cancer Therapy: An Update on Pharmacological Small-Molecule Compounds

**DOI:** 10.3389/fphar.2022.966012

**Published:** 2022-08-11

**Authors:** Lijuan Zhang, Yuxuan Zhu, Jiahui Zhang, Lan Zhang, Lu Chen

**Affiliations:** ^1^ Department of Pharmacy, Sichuan Academy of Medical Sciences & Sichuan Provincial People’s Hospital, School of Medicine, University of Electronic Science and Technology of China, Chengdu, China; ^2^ Personalized Drug Therapy Key Laboratory of Sichuan Province, School of Medicine, University of Electronic Science and Technology of China, Chengdu, China; ^3^ Sichuan Engineering Research Center for Biomimetic Synthesis of Natural Drugs, School of Life Science and Engineering, Southwest Jiaotong University, Chengdu, China; ^4^ School of Traditional Chinese Materia Medica, Key Laboratory of Structure-Based Drug Design and Discovery of Ministry of Education, Shenyang Pharmaceutical University, Shenyang, China

**Keywords:** autophagy, cytoprotective autophagy, small-molecule compound, inhibitor, cancer therapy

## Abstract

Autophagy is a self-degradation process in which damaged proteins and organelles are engulfed into autophagosomes for digestion and eventually recycled for cellular metabolism to maintain intracellular homeostasis. Accumulating studies have reported that autophagy has the Janus role in cancer as a tumor suppressor or an oncogenic role to promote the growth of established tumors and developing drug resistance. Importantly, cytoprotective autophagy plays a prominent role in many types of human cancers, thus inhibiting autophagy, and has been regarded as a promising therapeutic strategy for cancer therapy. Here, we focus on summarizing small-molecule compounds inhibiting the autophagy process, as well as further discuss other dual-target small-molecule compounds, combination strategies, and other strategies to improve potential cancer therapy. Therefore, these findings will shed new light on exploiting more small-molecule compounds inhibiting cytoprotective autophagy as candidate drugs for fighting human cancers in the future.

## Graphical Abstract

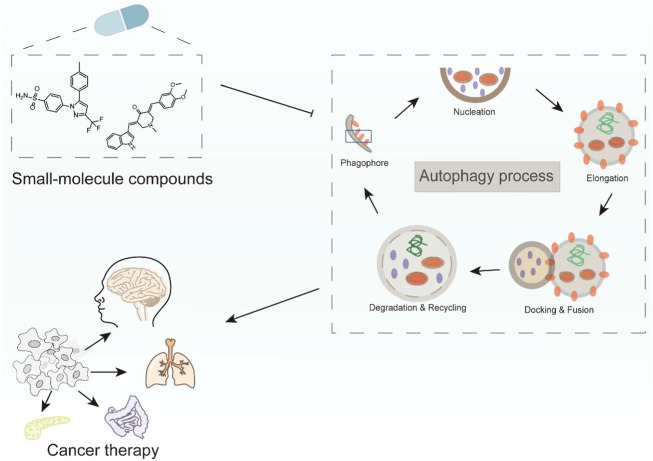

## Introduction

In eukaryotic cells, autophagy is an evolutionarily conserved process in which damaged or superfluous organelles, misfolded proteins, and invading microorganisms are degraded and removed by forming double-membrane autophagosomes and fusing with lysosomes (Mizushima, 2007). Autophagy has been reported to connect with multiple diseases, while the direct link between autophagy and cancer progress was proposed for the first time in 1999. In cancer cells, autophagy plays a dual role in promoting survival or inhibiting proliferation depending on the different contexts and stages of cancers (Singh et al., 2018). As a physiological process, autophagy can inhibit cancer by maintaining cell homeostasis, removing damaged organelles, and protecting normal cell growth. Once there is genetic damage that causes aberrant mutations, autophagy works to remove these aberrant mutations, thereby reducing the chance of cancer. (Barnard et al., 2016). In this case, the induction of autophagy by anticancer medications results in autophagic cell death acting as a cytotoxic process. On the contrary, as cancer progresses, autophagy is hijacked by cancer cells to provide the sufficient metabolic demands required for tumor survival and rapid proliferation. Thus, autophagy exerts a pro-survival mechanism contributing to cancer development and progression and protecting cancer cells from stress damage in advanced stages of cancer (Folkerts et al., 2019; Deng et al., 2020). Furthermore, autophagy involves in cancer cell resistance to chemotherapy/radiotherapy resulting in the mitigation of therapeutic effects (Das et al., 2018). Inhibition of cytoprotective autophagy sensitizes cancer cells to various treatments, enhancing the cytotoxicity of chemotherapeutic agents. Moreover, studies have reported that the genetic knockdown of autophagy-related genes (*Atgs*) or pharmacological inhibition of autophagy can effectively promote the programmed death of tumor cells in preclinical models (Pan et al., 2019).

Despite the complex interaction between suppressive and supportive roles of autophagy in cancer, the majority of autophagy inhibitors have been proposed as a strategy for improving cancer therapies and considered in some clinical trials. Previous studies have shown that patients with BRAF mutant melanoma regressed after conventional anti-BRAF and chloroquine combination therapy (Awada et al., 2022). These compounds have been identified as cytoprotective autophagy inhibitors by targeting the classical PI3K complex, such as 3-methyladenine (3-MA), Wortmannin, and LY294002, which inhibit the early stage of autophagy, and autolysosomes, like chloroquine (CQ), hydroxychloroquine (HCQ), and bafilomycin A1, which primarily inhibit the late stage of autophagy (Bao et al., 2018; Collins et al., 2018; Wu et al., 2021). However, clinical trials have revealed that autophagy inhibitors have a limited efficacy as mono-therapies. The combination of anticancer drugs and autophagy inhibitors contributes to the effect of chemotherapy. For example, the combination of HCQ and MEK inhibitor binimetinib significantly suppresses the tumor development in both organoid and patient-derived xenograft (PDX) models of pancreatic ductal adenocarcinoma (PDAC) (Bryant et al., 2019). Inhibiting autophagy enhances the cytotoxicity and anti-angiogenic ability of anlotinib, and co-administration of anlotinib and CQ reduces VEGFA levels in the tumor supernatant even more, compared with anlotinib or CQ treatment alone (Liang et al., 2019). Although numerous compounds have been identified as autophagy inhibitors, a few compounds other than CQ/HCQ are clinically available, most probably due to their toxicity and side effects due to the lake of specificity (Wang et al., 2016). Thus, the development of specific inhibitors targeting cytoprotective autophagy with potential clinical application becomes an urgent problem to be solved. In this review, we elaborate the molecular mechanisms that regulate autophagy and cytoprotective role of autophagy in cancer. Moreover, pharmacological small-molecule compounds blocking autophagy for cancer treatment are summarized, as well as combination therapies and dual-target small-molecule compounds involving autophagy modulators that can sensitize cancer cells to conventional therapies.

## The Cytoprotective Role of Autophagy in Cancer

### Mechanism and Process of Autophagy Induction

Autophagy mainly consists of four critical steps: initiation of autophagy, formation of autophagy, autophagosome docking and fusion, and autolysosome degradation (Li et al., 2020). The process of autophagy is controlled by a group of proteins encoded by the Atgs (Towers et al., 2020). Commonly, autophagy is triggered by nutrient deprivation or starvation condition, which results in Unc-51-like kinase1 (ULK1) complex activation. The ULK1 complex, consisting of ULK1, Atg13, FIP200, and Atg101, is negatively regulated by the mammalian target of rapamycin complex 1 (mTORC1) (Boya et al., 2013). Under nutrient starvation, mTORC1 is inhibited, which results in the subsequent induction of autophagy via dephosphorylating ULK1 (Zachari and Ganley, 2017). On the contrary, activated mTORC1 can phosphorylate ULK1 and suppress its kinase activity (Ganley et al., 2009). In addition to starvation, autophagy can also be activated by oxidative stress through the hypoxia-inducible factor 1-a (HIF-a). The accumulation of HIF-1a in hypoxic cells activates the BNIP/BNIP3L expression, which then dissociates the Bcl-2 and Beclin-1 (BECN1) complex to activate autophagy (Bellot et al., 2009). In the formation of autophagy, the ULK complex phosphorylates and activates the VPS34, part of PI3K complex including Beclin-1, VPS34, and other proteins, for preparing the membrane of the phagophore commonly from the endoplasmic reticulum (ER) (Behrends et al., 2010). In the final step of autophagy, autophagosome fuses with lysosomes to form autophagolyosome, and the cytoplasm-derived macromolecules can be degraded by enzymes in lysosomes (Li et al., 2020).

### The Functions of Cytoprotective Autophagy

Cytoprotective autophagy as a special type of autophagy plays a protective role in cancer cells via clearing damaged organelles, reducing DNA damage, and enhances the survival and resistance of the cancer cells, and finally promotes tumorigenesis and causes resistance to therapeutic agents. The process of cytoprotective autophagy provides energy and essential ingredients for cancer cells to survive via recycling cytoplasmic materials (Su et al., 2015; Vera-Ramirez et al., 2018). In addition, cytoprotective autophagy contributes to the aggressiveness of the cancers by facilitating metastasis (Macintosh et al., 2012; Vera-Ramirez et al., 2018). Therefore, cytoprotective autophagy exerts significant effect in cancer development; the inhibition of cytoprotective autophagy may be a promising approach for the treatment of cancers. In autophagy process, cytoprotective autophagy is regulated by several upstream signaling pathways. The mainly PI3K/AKT/mTOR pathway is alive at the level of aerobic glycolysis in cancer cells. In this pathway, mTOR is sensitive to nutritional signals, involved in mRNA translation control, protein transport, and degradation in cells and playing an important role in cell growth and proliferation (Al-Bari and Xu, 2020). AKT is a main PI3K effector to trigger the mTORC1 signaling pathway for autophagy inhibition, while mTORC2 can phosphorylate AKT on a critical target site in other cell types, suggesting a close interaction between PI3K and mTOR signaling. Ras-Raf–MEK1/2–ERK1/2 pathway is another key important signaling to regulate autophagy in cancer cells. PI3K and Ras are both stimulated by growth factor combining with receptor tyrosine kinases to produce class I PtdIns3K (PI3K-I) and GTPase Ras, respectively (Yaeger and Corcoran, 2019). Then, PI3K-I activates the AKT via increasing membrane recruitment of AKT and phosphoinositide-dependent protein kinase 1 (PDK1). Furthermore, AKT and ERK1/2 can activate mTORC1 through phosphorylating and inhibiting the tuberous sclerosis complex 1/2 (TSC1/TSC2), causing inhibition of autophagy. In addition, activated ERK1/2 also stimulates autophagy through directly targeting downstream protein of autophagy (Inoki et al., 2002; Ma et al., 2005). The AMP-activated protein kinase (AMPK) signaling is another regulating pathway of autophagy. The decrease of ATP/AMP ratio can activate the AMPK pathway through the upstream proteins including LKB1, CaMKKb, and TAK1 kinase. Then, active AMPK will phosphorylate and activate TSC1/TSC2, which lead to inactivation of mTORC1 and finally autophagy induction (Liang et al., 2007; Gwinn et al., 2008; Herrero-Martin et al., 2009). The p53 is a tumor suppressor playing dual roles in autophagy induction. In the aspect of nuclear p53, it can activate AMPK, which results in the activation of the TSC1/TSC2 complex and leading to the inhibition of the mTORC1 pathway and subsequent autophagy induction. On the contrary, cytoplasmic p53 exerts inhibitory function toward autophagy without mTOR (Feng et al., 2005; Tasdemir et al., 2008).

## Small-Molecule Compounds Inhibiting the Autophagic Process

**FIGURE 1:**
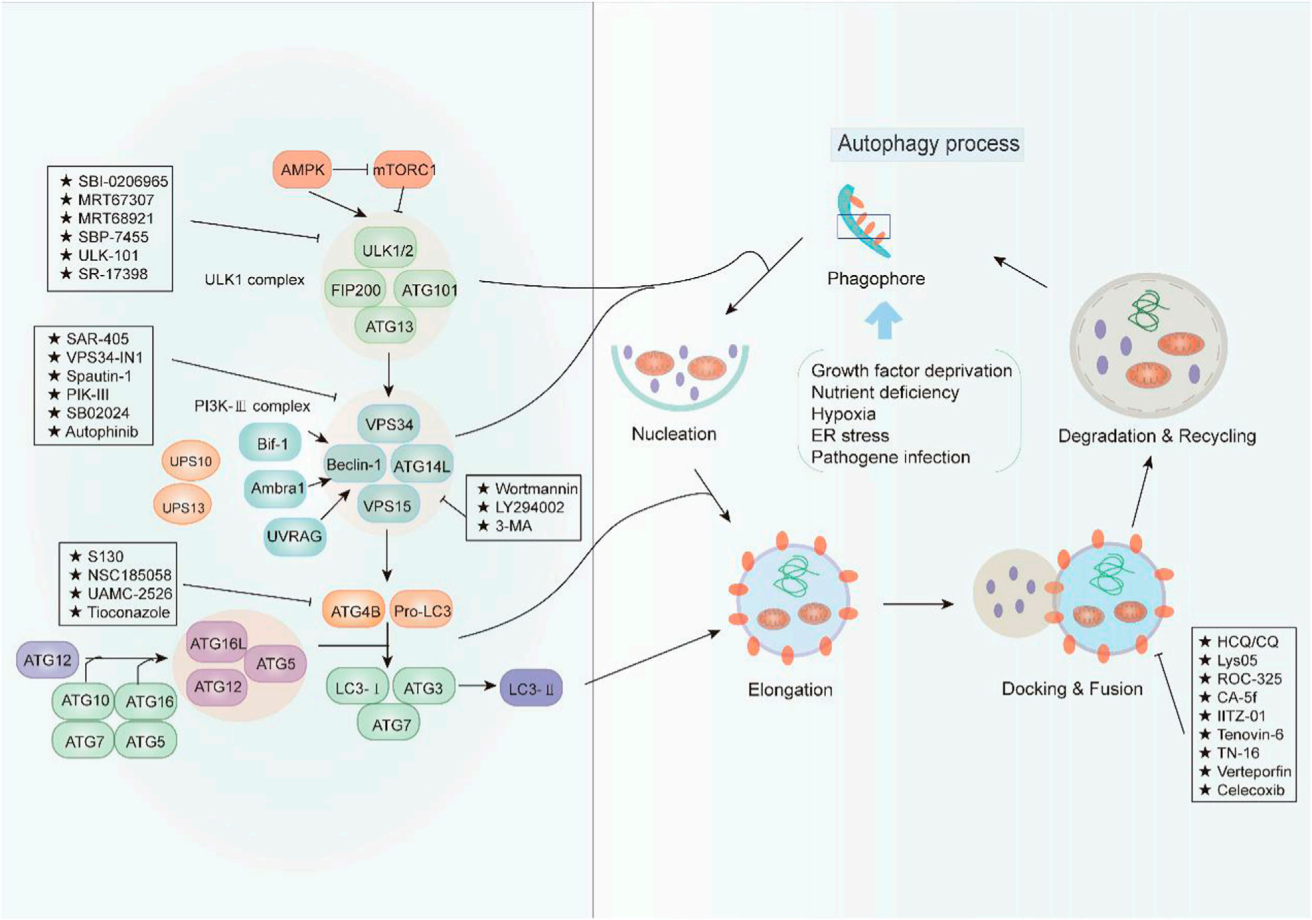
An overview of the modulation of autophagy. The initiation of autophagy is controlled by the ULK1 kinase complex that integrates stress signals from mTORC1 and AMPK. When mTORC1 kinase activity is inhibited, ULK1 is activated and binding with multiple ATG proteins like ATG101, ATG13, and FIP200 to engage the formation of phagophore. Then, the ULK1 complex activates the autophagosome formation by the phosphorylation of VPS34 and Beclin-1, which forms a PI3K-III complex. Beclin 1 interacts with factors (Ambra1, Bif-1, UVRAG, and ATG14L) that modulate its binding to VPS34 whose lipid kinase activity is essential for autophagy. Cellular concentrations of the initiation complex are also under the control of an ubiquitination cascade regulated by the deubiquitination peptidases USP10 and USP13. In addition, the LC3 system is required for autophagosome transport and maturation. In the autophagosome maturation, pro-LC3 could be cleaved by ATG4B with assistance of ATG3 and ATG7 as well as ATG5, ATG12, and ATG16. Mature autophagosomes fuse with lysosomes to degrade their cargo, and recycle essential biomolecules. Some small-molecule compounds can suppress autophagy by targeting early or late stages in the pathway.

Autophagy is a complex physiological process exerting a significant role in maintaining intracellular homeostasis. In view of the pro-survival effect of autophagy in cancer cells, cytoprotective autophagy inhibition should be greatly beneficial to cancer therapy. In recent years, an increasing number of compounds for autophagy inhibition have been discovered with great potential clinical application (Figure 1). According to their effect on the major steps of autophagy, we classified these small-molecule compounds and elucidated their potential mechanism in cancer treatment (Table 1).

**TABLE 1:** Small-molecule compounds for inhibiting autophagy in cancer therapy.

Target	Compound	Mechanism	Cancer	Biological activity	Ref.
ULK1/2 inhibitor	SBI-0206965	Inhibiting cytoprotective autophagy and promote apoptosis by destabilizing the pro-survival proteins Bcl-2/Bcl-xl	Lung cancer/triple-negative breast cancer	ULK1 IC_50_ = 108 nM	Xiang et al. (2020)
				ULK2 IC_50_ = 711 nM	
	MRT67307	Blocking ATG through targeting ULK1	Cancer	ULK1 IC_50_ = 45 nM	Chan, (2009)
				ULK2 IC_50_ = 38 nM	
	MRT68921	Inhibiting autophagy by reducing the transformation of LC3-I to LC3-II	High-grade serous ovarian cancer	ULK1 IC_50_ = 2.9 nM	Ren et al. (2020)
				ULK2 IC_50_ = 1.1 nM	
	SBP-7455	Inhibiting autophagy by targeting ULK1	Triple-negative breast cancer	IC_50_ = 13 nM	Tang et al. (2017)
	ULK-101	Inhibiting autophagy by targeting ULK1	Lung cancer	ULK1 IC_50_ = 8.3 nM	Petherick et al. (2015)
				ULK2 IC_50_ = 30 nM	
				EC = 390 nM	
	ULK-100	Inhibiting autophagy by targeting ULK1	Lung cancer	ULK1 IC_50_ = 1.6 nM	Petherick et al. (2015)
				ULK2 IC_50_ = 2.6 nM	
				EC = 83 nM	
	SR-17398	Inhibiting autophagy by targeting ULK1	Lung cancer	IC_50_ = 22.4 μM	Singha et al. (2020)
Non-selective PI3K Inhibitor	Wortmannin	Inhibiting autophagy by targeting PI3K	Colon cancer	IC_50_ = 20 nM	Zhang et al. (2020)
	3-Methyladenine	Inhibiting hypoxia-induced autophagy and increasing hypoxia-induced cell apoptosis	Cancer	IC_50_ = 60 μM	Ihle et al. (2004)
	LY294002	Inhibiting autophagy, inducing apoptosis and cell cycle arrest	Pancreatic cancer	IC_50_ = 0.5 μM	Wu et al. (2010), Wong et al. (2017), Dai et al. (2019), Andreidesz et al. (2021)
VPS34 inhibitor	SAR-405	Impeding autophagy through preventing autophagy vesicle trafficking	Renal tumor	IC_50_ = 1.2 nM	Yang and Klionsky (2010), Ohashi et al. (2019)
				KD = 1.5 nM	
	Compound 31	Inhibiting autophagy by targeting VPS34	Solid tumors	VPS34 IC_50_ = 2 nM	Pasquier, (2015)
				PI3Kα, β, δ, *γ* IC_50_ > 2 µM mTOR IC_50_ > 10 µM	
	VPS34-IN1	Impairing vesicular trafficking and mTORC1 signaling/inhibiting STAT5 phosphorylation downstream of FLT3-ITD signaling by targeting VPS34	Acute myeloid leukemia	IC_50_ = 1.2 Nm	Ronan et al. (2014), Pasquier et al. (2015)
				KD = 1.5 nM	
	Spautin-1	Activating GSK3β-induced apoptosis via inactivating PI3K/AKT pathway/suppressing melanoma growth via ROS-mediated DNA damage	Chronic myeloid leukemia/melanoma	IC_50_ = 0.45–1.03 μM	Bago et al. (2014), Shao et al. (2014), Meunier et al. (2020)
	PIK-III	Enhancing VPS34-dependence in cancer cells by impairing iron mobilization via the VPS34–RAB7A axis	Chronic myeloid leukemia	VPS34 IC_50_ = 18 nM mTOR IC_50_ > 9.1 μM	Liu et al. (2011), Guo et al. (2020)
	SB02024	Potentiating cytotoxicity of Sunitinib and Erlotinib in breast cancer cell/inducing an infiltration of NK, CD8^+^, and CD4^+^ T cells in melanoma and colorectal cancer	Breast cancer/colorectal cancer/melanoma	Kd = 4.5 μM	Dowdle et al. (2014), Kobylarz et al. (2020)
	Autophinib	Suppressing autophagy-mediated cell apoptosis via the AKT/mTOR pathway	Cancer	IC_50_ = 19 nM	Dyczynski et al. (2018)
ATG4B inhibitor	S130	Attenuating the delipidation of LC3 through targeting ATG4B to inhibit autophagy via PI3K/mTOR pathway	Colorectal cancer	IC_50_ = 3.24 µM	Nakatogawa et al. 2012)
				Kd = 4.0 µM	
	NSC185058	Attenuating the delipidation of LC3 through targeting ATG4B to inhibit autophagy	Osteosarcoma/breast cancer	IC_50_ = 51 µM	Bortnik et al. (2020)
	FMK-9a	Regulating cell autophagy through PI3K activation	Cervical cancer/glioblastoma	IC50 = 260 nM	Akin et al. (2014), Fu et al. (2019)
				Kd = 3.89 µM	
	UAMC-2526	Slowing down tumor growth and potentiating the effect of classical chemotherapy	Colorectal cancer	Plasma half-life = 126 min 70% metabolization after 30 min	Kurdi et al. (2017), Chu et al. (2018)
	Tioconazole	Suppressing autophagy and sensitizing cancer cells to chemotherapy	Breast cancer	ATG4A IC_50_ = 1.3 µM	Tanc et al. (2019)
				ATG4B IC_50_ = 1.8 µM	

### Small-Molecule Inhibitors of ULK1

**FIGURE 2:**
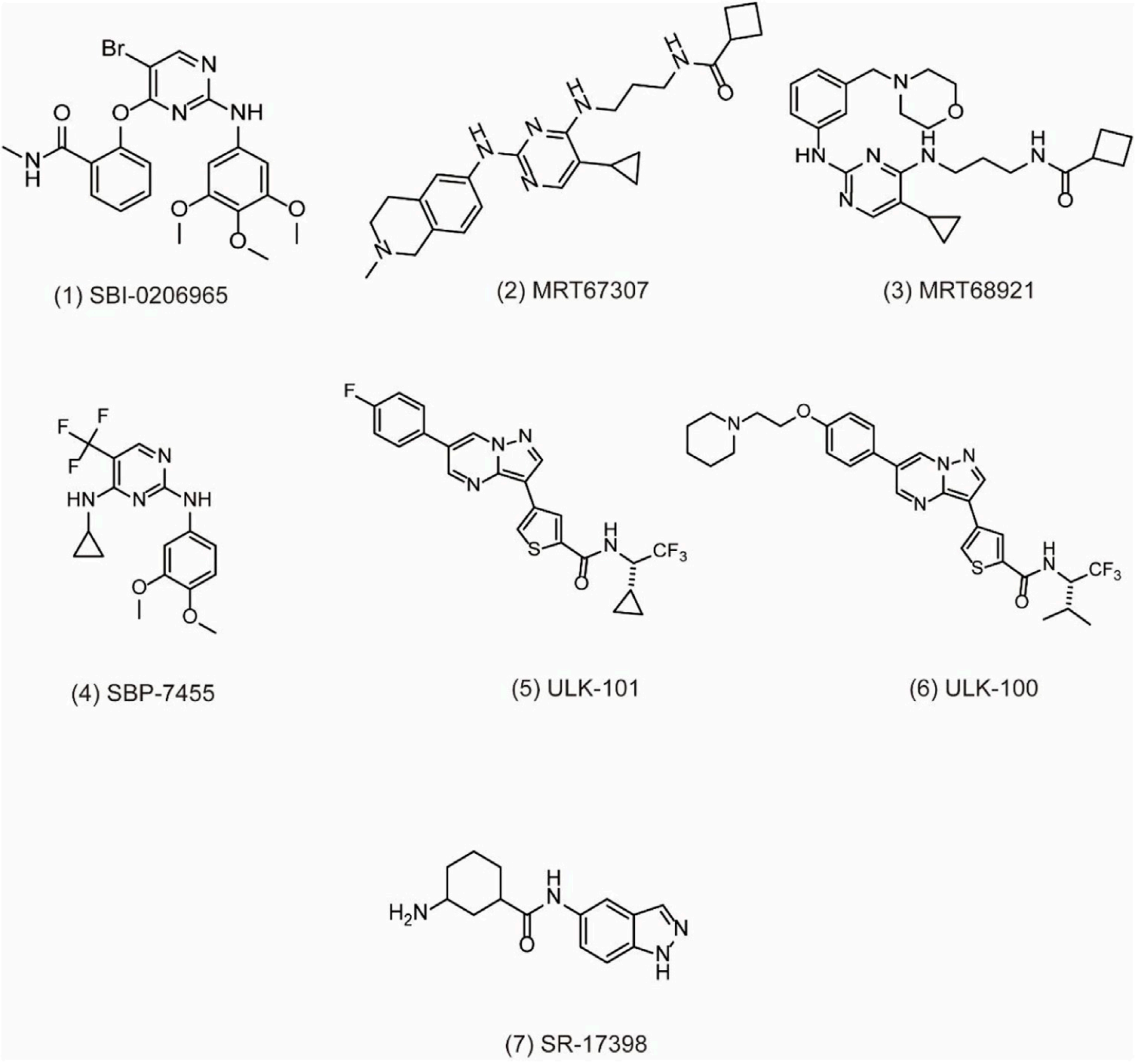
Chemical structures of **1–7** as inhibitors of autophagy by targeting ULK1.

Initially, ULK1 as a serine/threonine kinase plays one of the most important autophagy-related genes in human cells (Tsukada and Ohsumi, 1993; Xiang et al., 2020). Commonly, ULK1 is able to combine with other proteins including FIP200, Atg13, and Atg101 to form the ULK1 complex, which functions as a key regulator for autophagy initiation under the downstream of mTOR (Chan, 2009). Moreover, it has been reported that ULK2 is the functional homologue of ULK1, exerting a critical effect for autophagy regulation. Therefore, ULK1/2 is a key regulator in the formation of the phagophore, making it an interesting target for drug discovery. Based on the structure of ULK1/2, several inhibitors have been discovered. For example, SBI0206965 (Figure 2 **, 1)** is bound to the ATP binding site of ULK in the co-crystal structure of complex by forming a specific bidentate hydrogen bond between the trisubstituted pyrimidine scaffold and backbone residue Cys88 in the hinge region (Ren et al., 2020). SBI0206965 suppresses non-small cell lung cancer cell (NSCLC) growth by modulating both autophagy and apoptosis pathways. In depth, SBI0206965 attenuates the drug resistance of cisplatin in NSCLC by inhibiting cytoprotective autophagy and promotes apoptosis independent of autophagy by destabilizing the pro-survival proteins Bcl-2/Bcl-xL (Tang et al., 2017). In addition, MRT67307 **(** Figure 2 **, 2)** and MRT68921 (Figure 2 **, 3)**, previously identified as inhibitors of TANK binding protein 1 (TBK1), are also ULK1/2 inhibitors for their off-target effects on TBK1- and AMPK-related kinases. Both compounds require the key methionine 92 in the ATP binding pocket of ULK1 and probably disrupt the interaction between ULK1 and the scaffold proteins (Petherick et al., 2015). The experiment shows that these MRT compounds inhibit autophagy by reducing the transformation of LC3-I to LC3-II. Furthermore, MRT68921 is found to block ATG and suppress ovarian cancer development by targeting ULK1 kinase (Singha et al., 2020). In addition, a new optimized ULK1/2 inhibitor SBP-7455 (Figure 2 **, 4)** displays improved target binding affinity compared with SBI-0206965 and potently inhibits the ULK1/2 *in vitro* enzymatic activity, resulting in the reduction of viability in TNBC cells (Ren et al., 2020). Recently, two synthesized ULK1 inhibitors ULK-101 **(** Figure 2 **, 5)** and ULK-100 (Figure 2 **, 6)** also represent superior potency and selectivity to existing inhibitors in autophagy inhibition. Moreover, ULK-101 is demonstrated to sensitize KRAS mutant lung cancer cells to nutrient stress, indicating that nutrient-stressed cells may be particularly susceptible to ULK1 inhibition (Martin et al., 2018). SR-17398 **(** Figure 2 **, 7)** is a new inhibitor discovered by in silico high-throughput screen (HTS) and optimization of a series of ULK1 inhibitors. Further optimization of SR-17398 generates significantly better potency than the original structure (Wood et al., 2017). In consideration of the functions of ULK1 in autophagy, more work is needed to discover the specific ULK1 inhibitors for potential cancer therapeutic effects in animal models and clinical trials. The development of these more specific autophagy inhibitors provides for better anti-cancer treatment opportunities.

### Small-Molecule Inhibitors of PI3K

**FIGURE 3:**
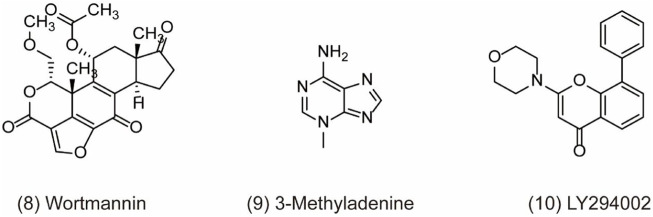
Chemical structures of **8–10** as inhibitors of autophagy by targeting PI3K-III.

Recently, accumulating studies about PI3K (phosphoinositide 3-kinases) inhibitors have enriched tumor treatment methods due to the indication of the relationship between the PI3K/Akt/mTOR pathway and drug resistance and prognosis of tumor. However, there are still many issues to be discussed in terms of drug mechanism, clinical trials, and efficacy and safety about PI3K inhibitors in the real world (Dey et al., 2017). As we know, PI3K is the family of intracellular lipid kinases that are unique to eukaryotic cells and usually divided into three categories based on their structural features and substrate specificities, including class I, class II, and class III PI3K (also known as VPS34/PIK3C3) (Yu et al., 2015). More interestingly, it has been reported that these three classes of PI3Ks play distinct roles in autophagy. Class I PI3K is demonstrated as the inhibitor of autophagy, which is contrary to class III PI3K for the induction of autophagy (Petiot et al., 2000). In a study, PIK3C3 can combine with BECN1 and PIK3R4 to form a protein complex and produce phosphatidylinositol 3-phosphate (PtdIns3P), which is essential for the initiation and development of autophagy (Zhang et al., 2020). Therefore, class III PI3K comes into the focus as a potent drug target for autophagy inhibition. For example, pan-PI3K inhibitors like wortmannin (PX-866) **(** Figure 3 **, 8)**, 3-methyladenine (3-MA) (Figure 3 **, 9)**, and LY294002 **(** Figure 3 **, 10)** are classical autophagy inhibitors. Wortmannin is derived from fungal metabolite and widely inhibits different classes of PI3K with good pharmacokinetics in human tumor xenografts. Wortmannin shows an anti-tumor activity both alone and in combination with anti-cancer drugs like cisplatin to sensitizing radiation treatment (Ihle et al., 2004). In addition, the analogue 17-hydroxy Wortmannin decreases drug resistance by restoring TRAIL’s response through ameliorating PIK3C3-beclin 1 (BECN1) complex and autophagy activity in colon cancer cells (Dai et al., 2019). However, 3-MA exerts a dual role in the modulation of autophagy via different temporal patterns of inhibition on class I and III PI3K (Wu et al., 2010). LY294002 is a synthetic compound like flavonoid quercetin ad can inhibit autophagy via competing with ATP to bind PI3K active sites. According to the research, LY294002 shows promising therapeutic effects on proliferation inhibition through inducing cell cycle arrest and apoptosis in various types of cancer (Wong et al., 2017; Peng et al., 2020; Andreidesz et al., 2021). In addition, LY294002 combining with anticancer drugs promotes an anticancer effect on breast cancer cells (Abdallah et al., 2020). However, pan-PI3K inhibitors lack specificity and usually results in an off-target effect on other PI3K-related kinases such as mTOR, finally inducing unfavorable side effects in clinical trials. Therefore, the development of potent selective PI3K inhibitors holds the limelight in autophagy research.

### Small-Molecule Inhibitors of VPS34

**FIGURE 4:**
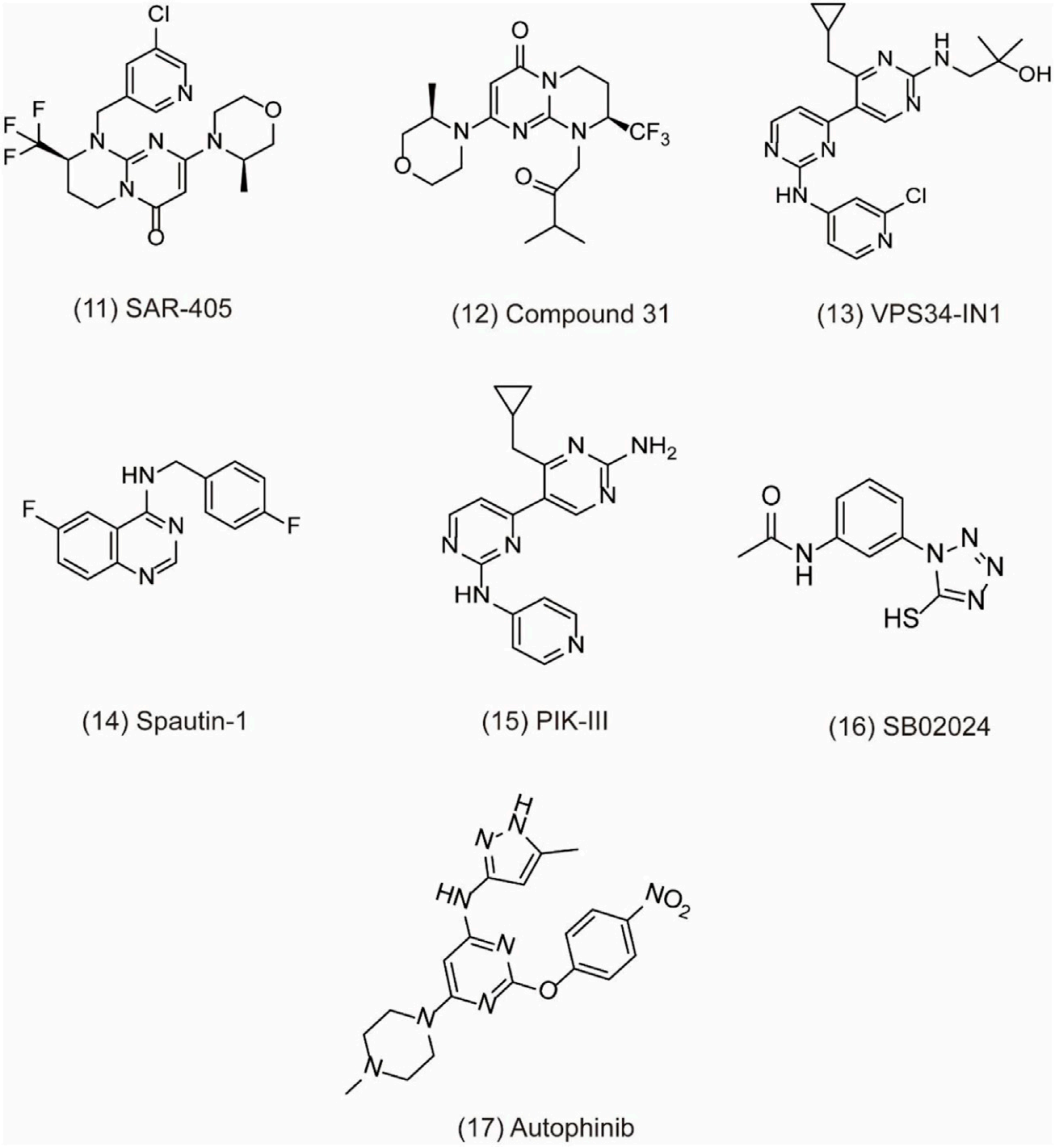
Chemical structures of **11–17** as inhibitors of autophagy by targeting VPS34.

VPS34 (vacuolar protein sorting 34) as a catalytic subunit of PI3K is originally discovered in yeast mutants and the only PI3K found in yeast (Yang and Klionsky, 2010). Based on the structure, VPS34 is mainly composed of C2 domain in the N-terminal, CCD domain, and phospholipid kinase domain in the C-terminal. The C2 domain can bind to beclin-1 without affecting the catalytic activity *in vitro*. However, the C-terminal is essential for its catalytic activity via suppressing the basal activity of the catalytic subunit. In addition, VPS34 regulates autophagosome synthesis and maturation by binding to Beclin1 and VPS15 as well as ATG14L/UVRAG to form three different PI3K complexes, which specifically phosphorylate the 3-hydroxyl of phosphatidylinositol (PI) to produce PIP_3_. Subsequently, PIP_3_ recruits PIP_3_ effector proteins containing FYVE or PX domains and regulates the formation of autophagosome membranes through a series of signal transduction (Ohashi et al., 2019). Therefore, the discovery of specific inhibitors for VPS34 may be potentially applied to treat cancer through autophagy pathway. For example, SAR405 **(** Figure 4 **, 11)** is a highly selective VPS34 inhibitor, exerting autophagy inhibition effect by preventing autophagy vesicle trafficking from late endosomes to lysosomes but reserving the function of early endosomes (Pasquier, 2015). In recent study, the combination of SAR405 with mTOR inhibitor everolimus results in synergistic anti-proliferative activity in renal tumor cell lines, indicating its potential clinical application in cancer (Ronan et al., 2014). Recently, a new synthetic pyrimidinone derivative named compound 31 ((2S)-8-[(3R)-3-methylmorpholin-4-yl]-1-(3-methyl-2-oxo-butyl)-2-(trifluoromethyl)-3,4-dihydro-2H-pyrimido [1,2-a] pyrimidin-6-one)) **(** Figure 4 **, 12)** also selectively inhibits VPS34. Based on the X-ray crystal structure in human VPS34, compound 31 specifically inhibits VPS34 due to its unique morpholine synthon (Pasquier et al., 2015). Another group of inhibitors are bis-aminopyrimidine derivatives including VPS34-IN1 **(** Figure 4 **, 13)** and PIK-III **(** Figure 4 **, 15)** by Novartis. VPS34-IN1 is found binding with the hydrophobic region of the kinase ATP domain to inhibit VPS34. Moreover, VPS34-IN1 inhibits autophagy in acute myeloid leukemia (AML) cells via impairing vesicular trafficking and mTORC1 signaling in connection with STAT5 phosphorylation, downstream of FLT3-ITD signaling (Meunier et al., 2020). Owing to the phosphatidylinositol 3-phosphate-binding SGK3 protein kinase as the downstream target of VPS34, the target may be a therapeutic strategy to treat tumor cells (Bago et al., 2014). Spautin-1 (MBCQ) **(** Figure 4 **, 14)** shows potent anticancer activity and improves the efficacy of imatinib mesylate by associating with GSK3β activation-induced apoptosis via inactivating the PI3K/AKT pathway in chronic myeloid leukemia (CML) (Shao et al., 2014). Moreover, Spautin-1 suppresses melanoma growth via damaging ROS-mediated DNA and promoting the degradation of PI3K complexes by inhibiting two ubiquitin-specific peptidases of USP10 and USP13 (Liu et al., 2011; Guo et al., 2020). PIK-III is also equipped with good selectivity over PIK3 and can be used to inhibit VPS34 enzymatic function with precision. PIK-III enhances VPS34-dependence in cancer cells by impairing iron mobilization via the VPS34-RAB7A (Kobylarz et al., 2020). In addition, NCOA4 is discovered as the substrate of PIK-III and can directly bind with ferritin heavy chain-1 (FTH1) to form an iron-binding ferritin complex for targeting autolysosomes (Dowdle et al., 2014). In addition, SB02024 **(** Figure 4 **, 16)** is an ATP competitive inhibitor and binds in the active site of VPS34 to inhibit its catalytic function. SB02024 efficiently inhibits autophagy and *in vitro or in vivo* cell viability while enhances its cytotoxic effect in breast cancer cell lines by combining with Sunitinib (Dyczynski et al., 2018). Additionally, inhibiting VPS34 induces the infiltration of NK, CD8^+^, and CD4^+^ T cells in melanoma and colorectal cancer (Noman et al., 2020). A novel potent VPS34 inhibitor named autophinib (Figure 4 **, 17)** is discovered by phenotypic screening to monitor small molecule that induce autophagy damage (Robke et al., 2017). Autophinib shows structural advantages with chloro-substituent at the 6-position and smaller replacement of the pyrazole in the 4-position and replacement of ether-oxygen in 2-position in the pyrimidine ring framework. With the emergence of these VPS34 specific inhibitors, autophagy inhibition has taken a new direction in cancer therapy, but further research is needed to reveal its role in tumor therapy.

### Small-Molecule Inhibitors of ATG4B

**FIGURE 5:**
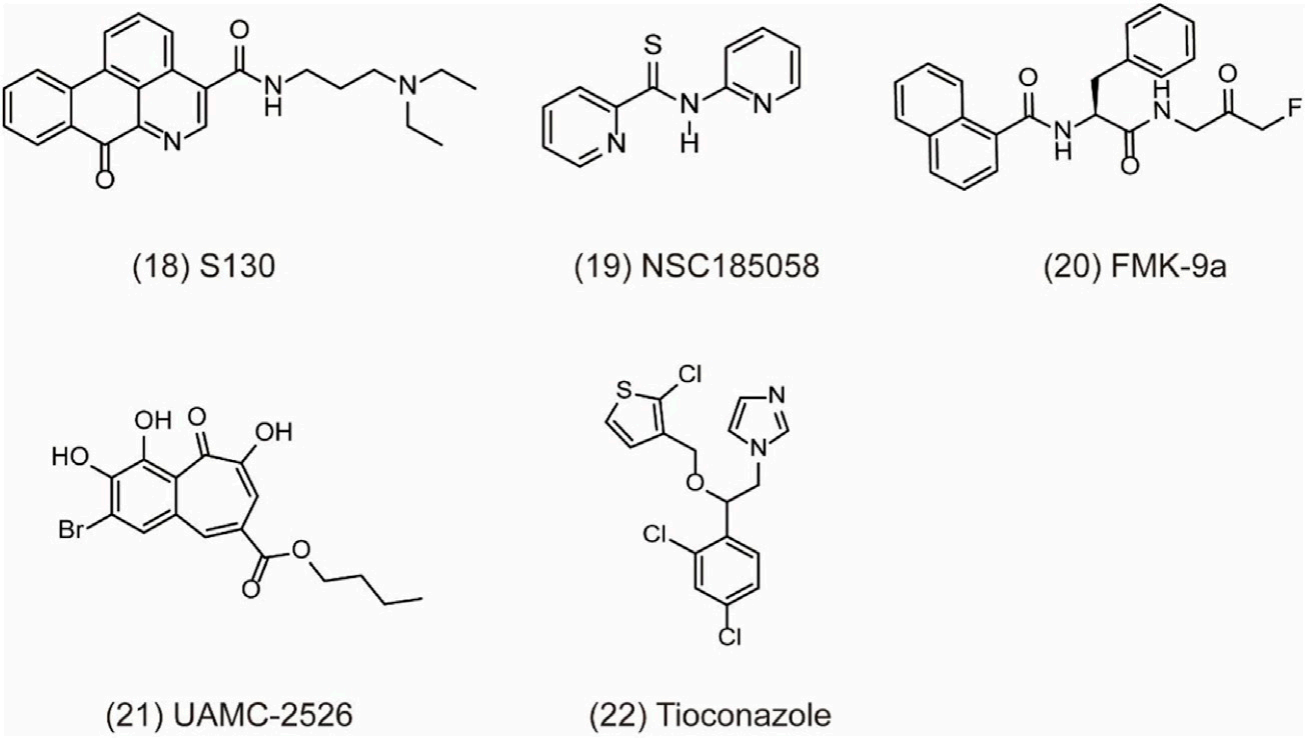
Chemical structures of **18–22** as inhibitors of autophagy by targeting ATG4B.

ATG4 is a pivotal autophagy-related cysteine protein family, which plays critical role in the autophagy process. Among the four homologs, ATG4B possesses superior efficiency. Hitherto, ATG4B has been discovered to form autophagic vesicles by promoting the activation of LC3-I through cleaving the C-terminus of proLC3 (ATG8 family member) and exposure to ATG7. Then, LC3-I can be conjugated to phosphatidylethanolamine by ATG3 to form LC3-II and recruited to the autophagosome membranes to promote autophagic vesicle growth and expansion (Li et al., 2011; Skytte Rasmussen et al., 2017). Finally, ATG4B can provide enough LC3 to sustain autophagy by recycling the lipid LC3 in cells (Nakatogawa et al., 2012). Therefore, ATG4B is a requisite enzymatic component in the autophagy process and is becoming increasingly attractive target for cancer therapy. However, the development of specific antagonists of Atg4B is still at a very preliminary stage. In-depth exploration of the interactions between Atg4B and LC3 is vital for the further discovery of more specific Atg4B antagonists. To date, increasing evidences confirm that ATG4B is overexpression in cancer cells, indicating that the ATG4B inhibition may be an effective approach for cancer treatment (Bortnik et al., 2020). Recently, considerable efforts have been made to identify small-molecule ATG4B inhibitors. For example, S130 **(** Figure 5 **, 18)** is a potent ATG4B inhibitor discovered by in silico screening and specifically suppresses the activity of ATG4B without affecting other proteases. S130 inhibits autophagy without causing the impairment of autophagosome fusion and dysfunction of lysosomes (Fu et al., 2019). Furthermore, S130 probably attenuates the delipidation of LC3-II to suppress the recycling of LC3-I. Therefore, S130 is an effective pharmacological agent to combine with ATG4B in autophagy for the treatment of cancer. NSC185058 **(** Figure 5 **, 19)** is the inhibitor derived from the NCI library. The mechanism of NSC185058 is like S130 to inhibit the delipidation of LC3, but not affect the mTOR or PI3K pathways. Furthermore, NSC185058 as an ATG4B antagonist can suppress tumor growth by autophagy inhibition (Akin et al., 2014). In addition, FMK-9a **(** Figure 5 **, 20)** is also a potential ATG4B inhibitor and plays multiple roles in autophagy process. FMK-9a blocks the activation of pro-LC3 and delipidation of LC3 via inhibiting ATG4B, but phenotypically initiates cell autophagy through PI3K activation (Chu et al., 2018). However, autophagy levels are still increased in FMK-9a-treated cells, suggesting that FMK-9a induces autophagy independent of ATG4B. In addition, one study showed that FMK-9a could not inhibit Hela cell growth; thus, it could not yet be studied as an anticancer compound. In another study, small molecules with a benzotropolone backbone structure will be an effective approach to enhance the sensitivity to chemotherapy and attenuate tumor growth by the impairment of autophagy through targeting ATG4B (Kurdi et al., 2017). UAMC-2526 **(** Figure 5 **, 21)** is the optimal compound for its fair plasma stability. This compound significantly inhibits tumor growth with the assistance of oxaliplatin and potentiates the chemotherapeutic effect of gemcitabine in pancreatic ductal adenocarcinoma (Tanc et al., 2019; Takhsha et al., 2021). Tioconazole **(** Figure 5 **, 22)** used as a clinical antifungal drug approved by FDA is discovered as an ATG4/ATG4B inhibitor. Based on computational docking and molecular dynamics (MD) simulations, tioconazole can stably occupy the active site of ATG4 and transiently interact with the allosteric regulation site in LC3 to inhibit autophagy flux (Liu et al., 2018). In a word, ATG4B is a core autophagy-related protein and shows promising direction for cancer therapy by inhibiting autophagy.

## Other Therapeutic Strategies Inhibiting Cytoprotective Autophagy

**FIGURE 6:**
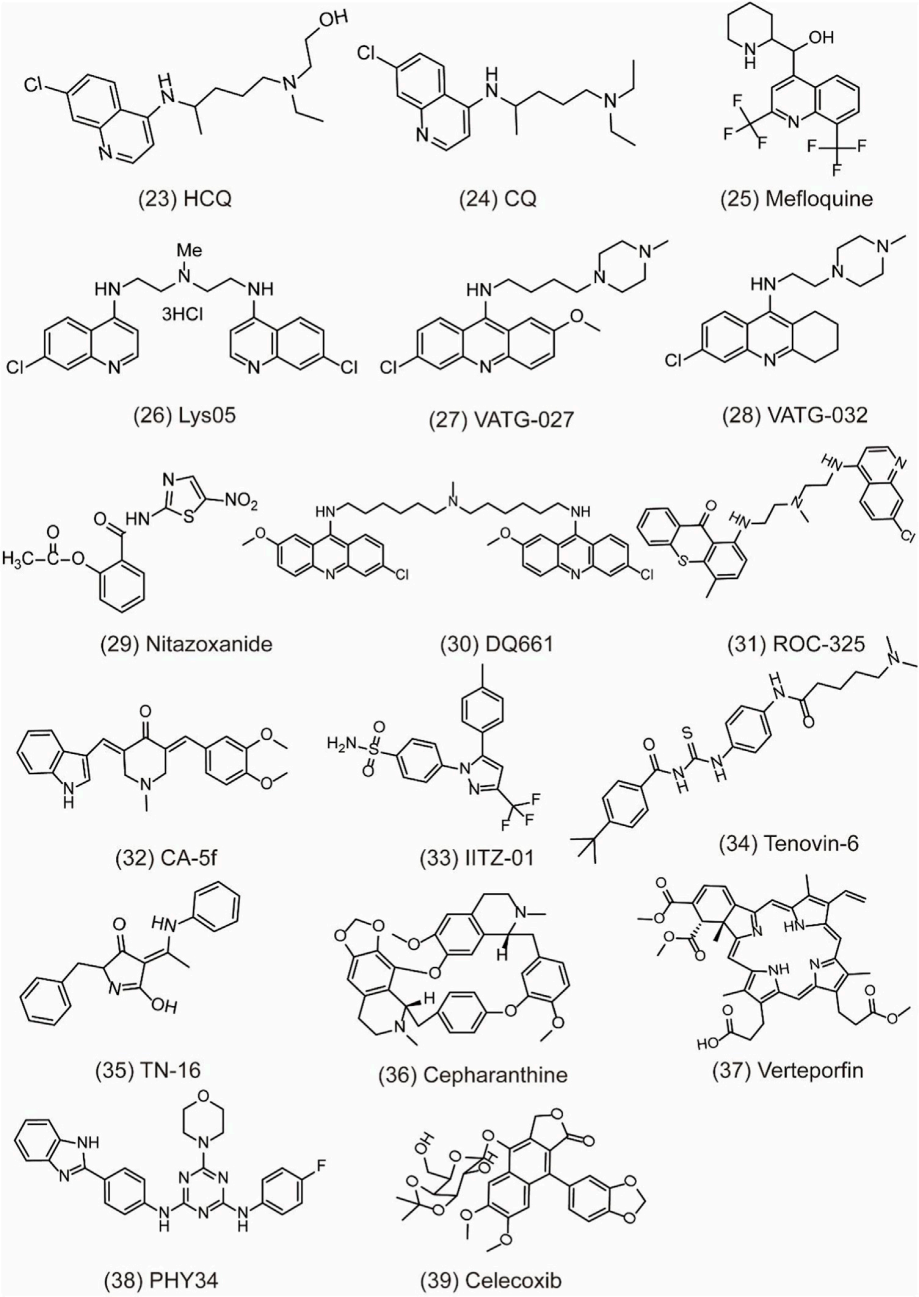
Chemical structures of **23–39** as inhibitors of autophagy by targeting other targets.

So far, there are little upstream autophagy inhibitors with well-defined targets successfully entering into clinical trials, but other inhibitors targeting proteins or pathways show the convincing *in vivo* activity (Table.2). For example, CQ/HCQ (Figure 6 **, 23) (** Figure 6 **, 24)** has been used clinically for cancer therapy by autophagy inhibition. CQ/HCQ mainly impairs autophagosome fusion with lysosomes due to the autophagy-independent severe disorganization of the Golgi and endo-lysosomal systems (Mauthe et al., 2018). At present, many clinical researchers continue to study chloroquine in various forms. Some scientists say this drug lacks specificity, but there are still many early trials underway. Moreover, there are plans to combine hydroxychloroquine with other anticancer drugs, which have shown promising results. For example, the combination of CQ and cisplatin (CDDP) can significantly increase cytotoxicity by decreasing proliferation and increasing cell apoptosis via the induction of p21^WAF1/CIP1^expression and autophagy inhibition in drug-resistant cells (Hwang et al., 2020). In another study, CQ is an effective adjunct to inhibit triple negative breast cancer (TNBC) cell metastasis and proliferation with carboplatin via targeting cancer stem cells (CSCs) that is the character of TNBC (Liang et al., 2016). Recently, other quinoline analogs are synthesized to improve their anti-cancer efficacy. For example, mefloquine (MQ) **(** Figure 6 **, 25)** shows the better potent efficacy in autophagy inhibition and produces unexpected anticancer effects in breast cancer (Sharma et al., 2012). Due to the limited effect of HCQ in clinical trials, the structural modification of CQ/HCQ is necessary. Synthesis of bisaminoquinoline can potently inhibit autophagy and impair *in vivo* tumor growth. In addition, CQ derivatives such as Lys01–Lys05 potently impair autophagy as the presence of two aminoquinoline rings and a triamine linker and C-7 chlorine (McAfee et al., 2012). Lys05 **(** Figure 6 **, 26)** suppresses autophagy by the phosphorylation of sequestosome-1 (SQSTM1/p62) and proline-rich AKT1 substrate 1 (AKT1S1) (Ondrej et al., 2020). In addition, a novel series of acridine and tetrahydroacridine derivatives are synthesized as autophagy inhibitors. For example, VATG-027 **(** Figure 6 **, 27)** and VATG-032 **(** Figure 6 **, 28)** have therapeutic potential to sensitize oncogenic BRAF V600E mutant melanoma tumor to vemurafenib by lysosomal deacidification and disruption of autophagosome (Goodall et al., 2014). Likewise, prodigiosin also sensitizes colorectal cancer cells to 5-fluorouracil by impairing autophagosome–lysosome fusion (Zhao et al., 2020). Nitazoxanide **(** Figure 6 **, 29)** is similar to VATGs in inhibiting autophagy through the blockage of late-stage lysosome acidification in glioblastoma (Wang et al., 2018). Moreover, a novel antimalarial named dimeric quinacrine 661 (DQ661) **(** Figure 6 **, 30)** inhibits autophagy by targeting palmitoyl-protein thioesterase 1 (PPT1) and exerts anticancer effect in various tumors (Rebecca et al., 2017). Therefore, lysosomal-directed PPT1 inhibitors represent a novel approach to concurrently targeting late-autophagosomal process in cancer. DQ661 is found to block autophagy more than hydroxychloroquine, which is important for improving the efficacy and reducing off-target toxicity. ROC-325 **(** Figure 6 **, 31)** is a very promising novel lysosomal autophagy inhibitor, exhibiting superior anticancer efficacy than HCQ in the preclinical study (Jones et al., 2019). The mechanism of ROC-325 is to antagonize renal cell carcinoma (RCC) growth and survival by inhibiting ATG5/7-dependent autophagic degradation and inducing apoptosis (Carew et al., 2017). Furthermore, the oral medication of ROC-325 exerts efficiency and is well tolerated in models of RCC rats (Carew and Nawrocki, 2017). A curcumin analog CA-5f **(** Figure 6 **, 32)** is identified as a novel late-stage autophagy inhibitor with a potent anti-tumor effect against non-small cell lung cancer (Zhang et al., 2019). The s-triazine analogs such as IITZ-01 and IITZ-02 also act as potent lysosomotropic autophagy inhibitors for TNBC treatment (Guntuku et al., 2019). Moreover, IITZ-01 **(** Figure 6 **, 33)** can potentiate TRAIL-induced apoptosis in cancer cells by DR5 upregulation and survivin downregulation via ubiquitin–proteasome pathway (Shahriyar et al., 2020). Tenovin-6 **(** Figure 6 **, 34)** also impairs the autophagy in chronic lymphocytic leukemia cells by affecting the acidification of autolysosomes and hydrolytic activity of lysosomes (Yuan et al., 2017). TN-16 **(** Figure 6 **, 35)** is also a potential autophagy inhibitor with the blockade of autophagosome–lysosome fusion at the later stage of autophagy (Hasanain et al., 2020). Other autophagy inhibitors like cepharanthine (CEP) **(** Figure 6 **, 36)**, verteporfin **(** Figure 6 **, 37)**, and PHY34 **(** Figure 6 **, 38)** also exert an effect on different cancer cells (Donohue et al., 2011; Tang et al., 2018; Young et al., 2018). Cepharanthine can enhance the anti-cancer property of dacomitinib (DAC) in non-small cell lung cancer by blocking the autophagosome–lysosome fusion and inhibiting lysosomal cathepsin B and cathepsin D maturation (Tang et al., 2018). Verteporfin suppresses basal and IFN-induced PD-L1 expression by disrupting multiple steps of autophagy and STAT1–IRF1–TRIM28 signaling axis (Liang et al., 2020). In a study, verteporfin sensitizes osteosarcoma cells to chemotherapy by inducing p53 and impairs ubiquitin proteasomal degradation pathway (UPS) by the accumulation of p62 under ROS stress (Saini et al., 2021). PHY34 significantly decreases tumor proliferation in ovarian cancer by inhibiting autophagy via targeting the ATP6V0A2 subunit of V (vacuolar)-ATPase and altering nuclear localization of proteins (Salvi et al., 2022). In addition, celecoxib **(** Figure 6 **, 39)** is used as an anti-inflammatory agent, but has been discovered to have the antitumor effect in HL-60 acute leukemia cells by affecting lysosome function (Lu et al., 2016). Bafilomycin A1 is also an inhibitor of late autophagy inhibitor by preventing the fusion of autophagosome and lysosome, as well as suppressing the degradation of protein in autolysosome (Fischer et al., 2020). Although these lysosomal inhibitors show positive *in vivo* effect, their mechanism remains unclear as a lack of clear target. Therefore, it is necessary to develop more potent, specific, and effective autophagy inhibitors for cancer treatment.

**TABLE 2:** Other small-molecule compounds for inhibiting autophagy in cancer therapy.

Compound	Mechanism	Cancer	Biological activity	Ref.
Hydroxychloroquine	Impairing autophagosome fusion with lysosomes	Breast cancer/pancreatic cancer/colon cancer/renal cancer/melanoma	IC_50_ = 15–42 μM	Takhsha et al. (2021)
Chloroquine	Impairing autophagosome fusion with lysosomes/increasing cytotoxicity by decreasing proliferation and inducing cell apoptosis via the induction of p21WAF1/CIP1expression and autophagy inhibition	Pancreatic adenocarcinoma/triple-negative breast cancer	EC = 15 µM	Liu et al. (2018), Mauthe et al. (2018)
Mefloquine	Inhibiting glioblastoma angiogenesis via disrupting lysosomal function/inhibiting NF-κB signaling and inducing apoptosis	Breast cancer/glioblastoma/colorectal cancer	EC = 0.5 µM	Hwang et al. (2020)
			EC/EC_CQ_ = 30	
Lys05	Suppressing autophagy by phosphorylating p62 and AKT1S1	Lung cancer	IC_50_ = 3.6 µM	Sharma et al. (2012), Liang et al. (2016)
VATG-027	Sensitizing melanoma tumor to vemurafenib by lysosomal deacidification and disruption of autophagosome	Melanoma	IC_50_ = 0.7 µM	McAfee et al. (2012); Ondrej et al. (2020)
			EC = 0.1 µM	
VATG-032	Sensitizing melanoma tumor to vemurafenib by lysosomal deacidification and disruption of autophagosome	Melanoma	IC_50_ = 27 µM	McAfee et al. (2012); Ondrej et al. (2020)
			EC = 5 µM	
Nitazoxanide	Blocking late-stage lysosome acidification	Glioblastoma	IC_50_ = 383.4–659.9 μM	Goodall et al. (2014)
Dimeric quinacrine 661 (DQ661)	Inhibiting autophagy by targeting palmitoyl-protein thioesterase 1 (PPT1)	Melanoma/pancreatic cancer	IC_50_ = 15 μM	Zhao et al. (2020)
ROC-325	Inhibiting ATG5/7-dependent autophagic degradation and inducing apoptosis	Renal cell carcinoma	IC_50_ = 4.9 µM	Rebecca et al. (2017), Wang et al. (2018), Jones et al. (2019)
CA-5f	Suppressing autophagosome–lysosome fusion/exhibiting strong cytotoxicity by increasing mitochondrial-derived reactive oxygen species (ROS) production	Lung cancer	IC_50_ = 20 μM	Carew et al. (2017)
IITZ-01	Potentiating TRAIL-induced apoptosis by DR5 upregulation and survivin downregulation via ubiquitin–proteasome pathway	Renal cancer/lung cancer/triple-negative breast cancer	IC_50_ = 2.6 μM	Carew and Nawrocki (2017), Zhang et al. (2019)
Tenovin-6	Affecting the acidification of autolysosomes and hydrolytic activity of lysosomes	Leukemia	IC_50_ = 9.6 ± 0.8 μM	Guntuku et al. (2019)
TN-16	Blocking autophagosome–lysosome fusion	Breast cancer	IC_50_ = 0.4–1.7 uM	Shahriyar et al. (2020)
Cepharanthine	Blocking autophagosome–lysosome fusion and inhibiting lysosomal cathepsin B and cathepsin D maturation	Non-small cell lung cancer/breast cancer	IC_50_ = 3.6 uM	Yuan et al. (2017)
Verteporfin	Inhibiting PD-L1 through autophagy and the STAT1–IRF1–TRIM28 signaling axis/inducing p53 and impairing ubiquitin proteasomal degradation pathway (UPS)	Pancreatic ductal adenocarcinoma/osteosarcoma	IC_50_ = 2.1–5.6 uM	Donohue et al. (2011), Young et al. (2018), Hasanain et al. (2020)
PHY34	Inhibiting autophagy by targeting the ATP6V0A2 subunit while interacting with cellular apoptosis susceptibility and altering nuclear localization of proteins	Ovarian cancer/breast cancer	HGSOC cell IC_50_ = 4 nM	Tang et al. (2018), Liang et al. (2020)
			MDA-MB-435 IC_50_ = 23 nM	
			MDA-MB-231 IC_50_ = 5.2 nM	
Celecoxib	Inhibiting cancer cell growth by modulating apoptosis and autophagy and reducing migration	Acute leukemia/osteosarcoma	IC_50_ = 40 nM	Saini et al. (2021)
Bafilomycin A1	Preventing the fusion of autophagosome and lysosome/suppressing the degradation of protein in autolysosome	Leukemia	IC_50_ = 4–400 nM	Salvi et al. (2022)

## Dual-Target Small-Molecule Compounds Inhibiting Cytoprotective Autophagy

**FIGURE 7:**
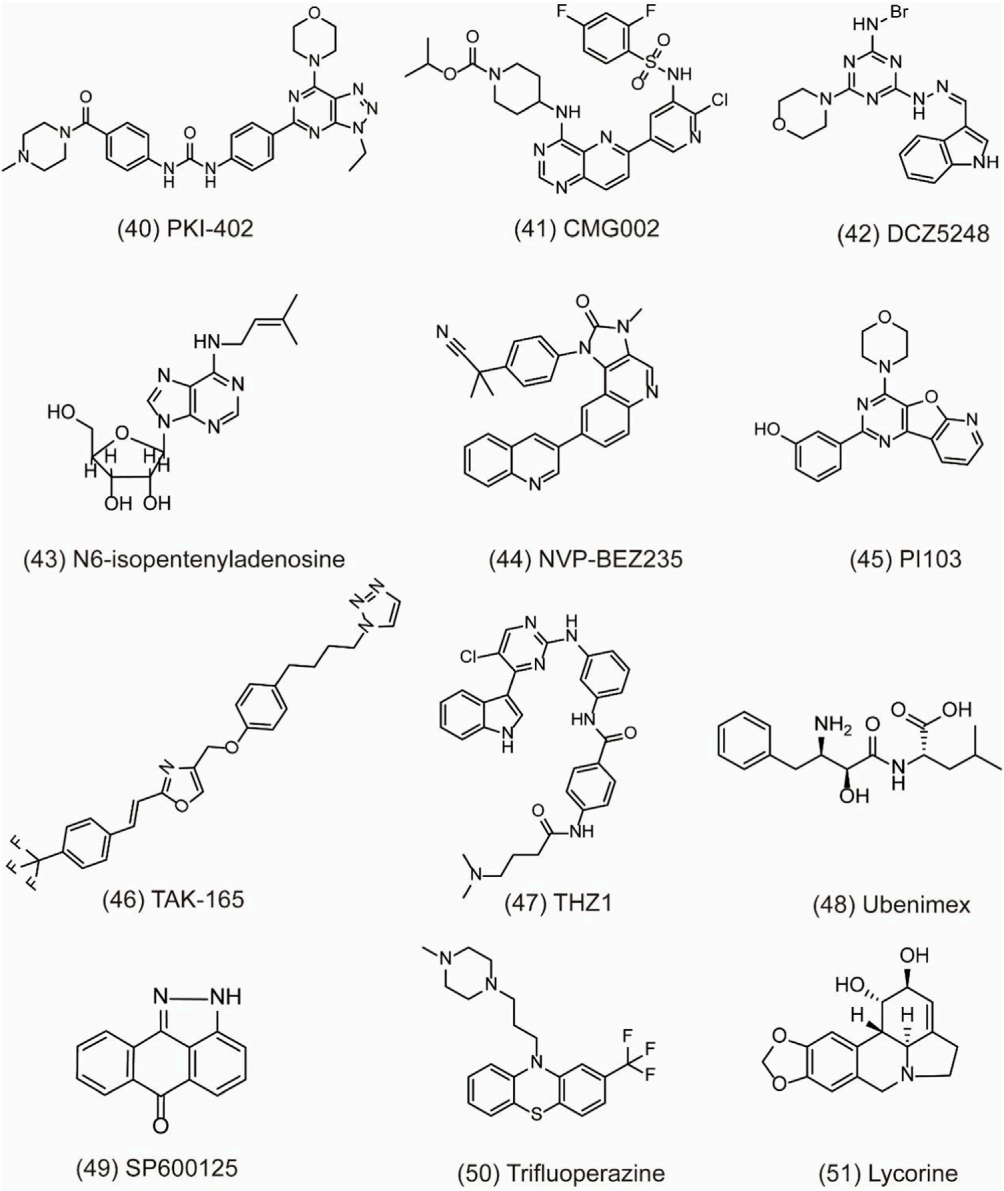
Dual-target and combination strategies of small-molecule compounds of **40–51** as inhibitors of autophagy.

Numerous autophagy inhibitors have been proved to exert different degrees of effect on tumor cells. However, the single-target compounds for cancers are not always aimed at the target protein and may develop the resistance to treatment. Thus, the occurrence of dual-target compounds and drug combination appears to be significant in tumor therapy **(** Table 3). The study indicates that the PI3K/AKT/mTOR signaling pathway is related to the cell proliferation, migration, and chemo-resistance. A few inhibitors can block both PI3K and mTOR to shut down the PI3K/Akt/mTOR pathway for the modulation of the autophagy process. For example, PKI-402 **(** Figure 7 **, 40)** as a dual PI3K/mTOR inhibitor improves chemo-resistance in ovarian cancer cells by depredating Mcl-1 protein and disrupting the balance of Bcl-2 family protein through autophagy inhibition (Hu et al., 2020). CMG002 **(** Figure 7 **, 41)** as a newly developed PI3K/mTOR dual inhibitor effectively induces gastric cancer cell death when combined with CQ by inducing the G0/G1 cell cycle arrest and enhancing apoptotic cell death in AGS and NUGC3 cells (Kim et al., 2019). Additionally, CMG002 significantly improves the chemo-resistance in ovarian cancer as well (Choi et al., 2019). DCZ5248 **(** Figure 7 **, 42)** is a novel dual inhibitor of Hsp90 and late-autophagy by inducing lysosomal acidification and lysosomal cathepsin activity. It shows antitumor activity by inducing the G1-phase cell cycle arrest and apoptosis in colon cancer cells. Therefore, DCZ5248 indicates that inhibiting both Hsp90 and autophagy provides a new therapeutic strategy for cancer treatment (Chen X. L. et al., 2021). N6-isopentenyladenosine (iPA) **(** Figure 7 **, 43)** is a novel autophagic flux inhibitor, exerting an anti-proliferative activity in melanoma cells by dual targeting AMPK and Rab7 prenylation. Furthermore, iPA induces autophagosome accumulation through activating AMPK to block mTOR pathway and impairs autophagic flux by blocking the autophagosome–lysosome fusion through the defective function of Rab7 (Ranieri et al., 2018).

**TABLE 3:** Dual and multiple targeted small-molecule compounds for inhibiting autophagy in cancer.

Target	Compound	Mechanism	Cancer	Biological activity	Ref.
PI3K/mTOR inhibitor	PKI-402	Suppressing cancer cell growth by degradation of Mcl-1 protein and disruption of the balance of Bcl-2 family protein	Ovarian cancer	IC_50_ = 2–16 nM	Lu et al. (2016)
PI3K/mTOR inhibitor	CMG002	Inducing G0/G1 cell cycle arrest and enhancing apoptotic cell death	Gastric cancer	AGS IC_50_ = 1.6 uM	Fischer et al. (2020), Hu et al. (2020)
				NUGC3 IC_50_ = 4.9 uM	
Hsp90 and late-autophagy inhibitor	DCZ5248	Inducing lysosomal acidification and lysosomal cathepsin activity/inducing G1-phase cell cycle arrest and apoptosis	Colon cancer	IC_50_ = 0.5 uM	Kim et al. (2019)
AMPK and Rab7 prenylation inhibitor	N6-isopentenyladenosine (iPA)	Impairing autophagic flux by blocking autophagosome–lysosome fusion through the defective function of Rab7	Melanoma	IC_50_ = 2.5 μM	Choi et al. (2019)
PI3K/mTOR inhibitor	NVP-BEZ235	Sensitizing cancer cells to radiotherapy through G2/M arrest and apoptotic cell death	Glioblastoma multiforme/thyroid cancer	IC_50_ = 38.9 nM	Ranieri et al. (2018), Chen X. L. et al. (2021)
PI3K/Akt/mTOR inhibitor	PI103	Inducing apoptosis, reducing autophagy, suppressing NHEJ and HR repair pathways in prostate cancer	Cancer	IC50 = 30 nM	Zhu et al. (2013)
HER2 inhibitor	TAK-165	Inhibiting autophagy in a HER2-independent manner	Acute myelocytic leukemia	IC50 = 6 nM	Chang et al. (2013)
CDK7 inhibitor	THZ1	Enhancing cytotoxicity via autophagy suppression	Renal cell carcinoma	IC50 = 3.2 nM	Chang et al. (2014)
CD13 inhibitor	Ubenimex	Sensitizing cancer cells to CDDP by autophagy through perturbing the CD13/EMP3/PI3K/AKT/NF-κB axis	Gastric cancer	IC50 = 20 μM	Huang et al. (2019)
JNK inhibitor	SP600125	Sensitizing cancer cells to oxaliplatin by inhibiting autophagy	Colorectal cancer	IC50 = 40 nM	Chen H. et al. (2021)
Lysosome inhibitor	Trifluoperazine	Inhibiting autophagy flux by impairing lysosomes acidification and decreasing protein level of cathepsin L	Glioblastoma	IC50 = 15 μM	Guo et al. (2019)
MEK/ERK inhibition	Lycorine	Enhancing the degradation of high mobility group box 1 (HMGB1)/suppressing MEK-ERK pathway and increasing Bcl-2–Beclin-1 interaction	Multiple myeloma	IC50 = 20 μM	Vasilevskaya et al. (2016)

## Combination Strategies of Small-Molecule Compounds Inhibiting Cytoprotective Autophagy

NVP-BEZ235 (Figure 7 **, 44)** is a novel dual PI3K/mTOR inhibitor developed by Novartis Pharma. Preclinical studies have indicated that NVP-BEZ235 can induce autophagic cell death when autophagy inhibits the survival of cancer cells. However, in advanced tumors, NVP-BEZ235 alone shows pro-survival effect but is totally reversed when combining with the autophagy inhibitor 3-MA. Therefore, the co-treatment of NVP-BEZ235 and autophagy inhibitors in cancer therapy by inhibiting autophagy is well documented. Notably, NVP-BEZ235 has been shown to sensitize cancer cells to radiotherapy through the G2/M arrest and apoptotic cell death (Chang et al., 2013; Zhu et al., 2013). PI103 **(** Figure 7 **, 45)** is also a dual PI3K/mTOR inhibitor, sensitizing radio-therapy by inducing apoptosis, reducing autophagy, and suppressing NHEJ and HR repair pathways in prostate cancer (Chang et al., 2014). In addition, AC220 (Quizartinib), an FLT3 receptor tyrosine kinase inhibitor, is developed to treat AML in clinical trials. Through virtual screening, TAK-165 (Mubritinib) **(** Figure 7 **, 46)**, an autophagy inhibitor, is identified as a synergistic drug with AC220 at low doses. Further study indicates that a combination of TAK-165 with AC220 can potently induce cancer cell death by HER2-independent autophagy (Ouchida et al., 2018). THZ1 **(** Figure 7 **, 47)** used as a cyclin-dependent kinase 7 (CDK7) inhibitor reduces cell viability and induces apoptosis in human renal cell carcinoma. The combination of THZ1 and temsirolimus can potently enhance cytotoxicity via autophagy suppression (Chow et al., 2020).

Particularly, some miRNAs can alter autophagy activities by regulating its downstream signaling like PI3K/AKT pathway and multiple autophagy regulators including Beclin-1, ATG5, ATG4B, and p62 (Huang et al., 2019). Autophagy modulation by miRNAs can sensitize cancer cells to chemotherapy and radiotherapy. Therefore, the discovery of miRNA regulators to inhibit autophagy is a novel and effective strategy for cancer treatment. In a study, peptidyl arginine deiminase 4 (PAD4) is identified as nasopharyngeal carcinoma (NPC) biomarker, negatively regulated by microRNA 3,164 (miR-3164). LINC00324 interacts with miR-3164 to modulate the PI3K/AKT pathway and suppress apoptosis and autophagy in carcinoma (Chen H. et al., 2021). CD13 is a transmembrane glycoprotein with metalloproteinase activity. It overexpresses in gastric cancer cells to improve cell invasion ability. Ubenimex **(** Figure 7 **, 48)** as a CD13 inhibitor overcomes the resistance of cisplatin (CDDP) and improves the sensitivity of GC cells to CDDP by autophagy through perturbing the CD13/EMP3/PI3K/AKT/NF-κB axis (Guo et al., 2019). JNK belonging to the family of MAPK can regulate the activation of stress signaling to induce hypoxia-induced autophagy. Therefore, inhibiting JNK1 suppresses hypoxia-induced autophagy and sensitizes cancer cells to chemotherapy in HT29 cells. SP600125 **(** Figure 7 **, 49)** is a JNK inhibitor and can sensitize HT29 cells to oxaliplatin by inhibiting autophagy (Vasilevskaya et al., 2016). Trifluoperazine (TFP) **(** Figure 7 **, 50)** used as an antipsychotic drug treats cancer by enhancing the radio-sensitivity in glioblastoma (GBM) via impairing homologous recombination. Further study indicates that the treatment with TFP inhibits autophagy flux by impairing lysosome acidification and decreasing the protein level of cathepsin L, contributing to the radio-resistance of GBM cells (Zhang et al., 2017). In addition, lycorine **(** Figure 7 **, 51)** is also an effective autophagy inhibitor and shows potential therapeutic effect alone or in combination with bortezomib on multiple myeloma. Treatment with lycorine enhances the degradation of high mobility group box 1 (HMGB1), an important regulator of autophagy. MEK–ERK activation is then suppressed, and Bcl-2–Beclin-1 interactions are increased, leading to the inhibition of pro-survival autophagy and cell death (Roy et al., 2016).

In conclusion, pro-survival autophagy is the main cause of drug resistance and radiotherapy tolerance and severely impedes the tumor treatment in clinics. Therefore, numerous autophagy inhibitors have been studied for their anticancer potential. Aiming at the key protein targets in autophagy, plenty of effective anti-tumor autophagy inhibitors have been developed. In addition, some novel inhibitors targeting regulators of autophagy-related pathways are also regarded as a promising strategy. The dual-targeted drugs and drug combination also exert antitumor ability in cancer cells with potent cytotoxicity. In a word, discovery of novel autophagy inhibitors is valuable to cancer therapy, and more strives should been given to elucidate antitumor mechanism in the future.

## Conclusion and Perspectives

As a highly conserved biological process, autophagy displays a dual role in cancer cells. In this review, we emphasize the pivotal role of autophagy as a cytoprotective and drug-resistant mechanism and propose the feasibility of research on autophagy inhibition in cancer treatment. Small-molecular compounds targeting key proteins in the autophagy process have been regarded as a promising strategy in tumor treatment. However, much of the studies focused on repositioning the antimalarial drug chloroquine and its derivatives, which are indirect autophagy inhibitors that are inexpensive, safe, and readily available for clinical trials. Studies show that when these agents are given in the right combinations, they can produce dramatic results. For example, CQ is found to improve vemurafenib sensitivity in resistant cell lines and provided for the first time in a patient harboring the V600E mutation (Levy et al., 2014). Additionally, a combination of CQ and gemcitabine enhances the antitumor activity for gallbladder cancer (Wang et al., 2020). Yet, other studies indicate that CQ and its derivative sensitization appears to be independent of autophagy inhibition as it offers only limited autophagy modulation. For example, a combination of ERK and autophagy inhibition is a treatment approach for pancreatic cancer. ERK inhibition enhances pancreatic ductal adenocarcinoma (PDAC) dependence on autophagy by the upregulation of autophagic processes, and autophagy inhibition enhances the ability of ERK inhibitors to mediate antitumor activity in KRAS-driven PDAC (Bryant et al., 2019). Therefore, the development of more selective and potent autophagy inhibitors must be essential to definitively promote the therapeutic benefit of targeting autophagy in cancer patients.

Currently, increasing compounds are developed to block the autophagy process by targeting the specific key proteins including ULK1 kinase, PI3K complex, VPS34, and ATG4B. Moreover, other compounds also contribute to the suppression of cytoprotective autophagy. In addition, the drug combination and dual-targeted drugs can also promote sensitization of radio- or chemo-therapy. Importantly, additional factors functioning in autophagy process can also be considered as potential targets to inhibit autophagy and overcome therapy resistance, such as MAPK and AKT. Therefore, the inhibition of cytoprotective autophagy to enhance the therapeutic benefit of current anticancer therapies is an area for intense investigation. However, there still exist some challenges for these compounds in patient treatment, which greatly hinder the application of autophagy inhibitors in cancer treatment. Firstly, almost all of autophagy-related genes have non-autophagic functions resulting in hindrance to the study of the related mechanism (Xiao et al., 2021). To overcome this obstacle, the further mechanism of autophagy should be elaborated clearly and more endeavors should be put on the hunting of high-selective and effective compounds. In addition, autophagy appears to both activate and inhibit cell senescence, but long-term inhibition of autophagy seems to permanently increase the risk of cancer. Therefore, a novel strategy of drug combination between an inhibitor of autophagy with chemotherapy shows obvious clinical outcome in patients. Finally, several compounds remain unknown for their specific target in autophagy, which is also worthy of subsequent works for target recognition.

Previously, scientists have been trying to elucidate the role of autophagy in cancer, finding that the process can both inhibit the formation of new tumors and promote the growth of existing ones. These findings lead to dozens of trials combining chloroquine or hydroxychloroquine, with radio-/chemo-therapy drugs and targeted anticancer drugs for patients with refractory skin, brain, blood, and other cancers. But the results are not so good. Particularly, autophagy inhibitors have been shown to inhibit RAS-driven cancer growth, but this finding has been difficult to replicate in subsequent experiments. Therefore, studies for small-molecule inhibitors of autophagy, such as Sanofi’s VPS34 inhibitor SAR405 and Millennium’s ATG7 small-molecule modulator, have been halted. Notably, growing evidences indicate that autophagy inhibition can also limit cancer growth through its effects on the tumor microenvironment and host immunity. Autophagy is a key immune escape mechanism; autophagy inhibitors promote inflammatory responses in the tumor micro-environment to enhance immune surveillance. Therefore, autophagy pathway is highly druggable and has multiple drug targets. In conclusion, autophagy inhibition is gaining focus as a potentially new therapeutic approach in cancer. The outlook for the clinical development of novel inhibitors that target upstream modulators of autophagy or lysosomal agents is studied right now, and small-molecule inhibitors are still promising for cancer treatment.

## Abbreviations

Atg, autophagy-related gene; 3-MA, 3-methyladenine; CQ, chloroquine; HCQ, hydroxychloroquine; PDK1, phosphoinositide-dependent protein kinase 1; mTOR, mammalian target of rapamycin; ULK1, Unc-51-like kinase 1; ER, endoplasmic reticulum; PI3K, phosphoinositide 3-kinases; CML, chronic myeloid leukemia; TNBC, triple negative breast cancer; CSCs, cancer stem cells; RCC, renal cell carcinoma; PPT1, palmitoyl-protein thioesterase 1; PAD4, peptidyl arginine deiminase 4; HMGB1, high mobility group box 1.
